# ASO Author Reflections: Two Decades of Progress in Curative Treatment of Locally Recurrent Rectal Cancer

**DOI:** 10.1245/s10434-026-20584-6

**Published:** 2026-09-11

**Authors:** F. E. C. Vande Kerckhove, D. M. J. Creemers, E. Banken, F. Piqeur, S. H. J. Ketelaers, R. R. J. Coebergh van den Braak, J. Tolenaar, R. J. Schipper, G. A. P. Nieuwenhuijzen, W. Setz-Pels, T. R. van Oudheusden, A. W. Daniëls-Gooszen, S. G. van Ravensteijn, I. E. G. van Hellemond, A. E. Verrijssen, H. M. U. Peulen, J. G. Bloemen, J. Nederend, H. J. T. Rutten, J. W. A Burger

**Affiliations:** 1 Department of Surgery, Catharina Hospital Eindhoven, Eindhoven, the Netherlands; 2 GROW Research Institute for Oncology and Reproduction, Maastricht University, Maastricht, the Netherlands; 3 Department of Medical Oncology, Catharina Hospital Eindhoven, Eindhoven, the Netherlands; 4 Department of Radiation Oncology, Catharina Hospital Eindhoven, Eindhoven, the Netherlands; 5 Department of Radiology, Catharina Hospital Eindhoven, Eindhoven, the Netherlands

## Past

Despite advances in diagnosis and treatment, locally recurrent rectal cancer (LRRC) remains a complex disease associated with poor oncological outcomes. Over the past two decades, important developments in high-resolution imaging, neoadjuvant treatment, perioperative care, and the centralization of care have transformed the management of LRRC. However, it has remained unclear whether these incremental advances have translated into measurable improvements in outcomes for patients undergoing curative-intent treatment. Accordingly, this study aimed to evaluate long-term trends in the management and oncological outcomes for patients with LRRC treated in a Dutch tertiary referral centre.

## Present

Our study included 560 patients who underwent curative-intent resection for a first LRRC between 2000 and 2023.^1^ Despite an increasingly complex case mix, with more patients presenting with comorbidities, multifocal disease, and synchronous metastases, oncological outcomes improved over time.^1^ The R0 resection rate increased from 59% to 78%, while cumulative sum analyses demonstrated progressive improvements in both overall and disease-free survival.^1^ Overall, outcomes for patients undergoing curative-intent treatment for LRRC have steadily improved over the past two decades. This may reflect advances in diagnostics, neoadjuvant therapy, surgical technique and perioperative care, centralization of care, and patient selection.

## Future

Although oncological outcomes after curative-intent treatment have improved substantially, a substantial group of patients with LRRC remain ineligible for curative-intent treatment.^2–4^ Further progress will depend not only on advances in multimodal therapy, but also on improving patient selection. Integrating advanced imaging with clinical and biological markers may better distinguish favourable from aggressive tumour biology, enabling more informed selection of patients for neoadjuvant treatment and surgery. Ongoing prospective studies, including PelvEx II,^5^ will clarify the role of induction chemotherapy and help determine which patients derive the greatest benefit from intensified multimodal treatment.
